# Synergistic protection of borate and silicate salts composite for controlling the chloride-induced pitting and uniform corrosion of steel reinforcement bars embedded in mortars

**DOI:** 10.1038/s41598-024-57485-1

**Published:** 2024-03-25

**Authors:** Raja Rizwan Hussain, Abdulrahman Alhozaimy, Abdulaziz Al-Negheimish, D. D. N. Singh, Mshtaq Ahmed

**Affiliations:** 1 Center of Excellence for Concrete Research and Testing (CoE-CRT), Civil Engineering Department, College of Engineering, King Saud University, PO Box: 800, 11421 Riyadh, Saudi Arabia; 2 Corrosion and Surface Engineering CSIR, National Metallurgical Laboratory, Jamshedpur, India; 3 College of Engineering, King Saud University, Riyadh, Saudi Arabia

**Keywords:** Steel rebars, Mortar, Pitting corrosion, Anti-corrosion materials, Borate-silicate, Passive layer, Nanotechnology, Chemistry, Engineering, Materials science, Nanoscience and technology

## Abstract

In this study, the efficacy of the combined effect of borate and silicate alkali metal salts added to mortars for controlling the chloride-induced uniform and localized corrosion of embedded steel rebars is examined. The individually added salts in mortars are found to have insignificant effects in terms of reducing the uniform corrosion rate and localized damage. However, their combination (0.50% sodium tetra borate + 0.10% sodium silicate added with respect to the weight of the binder) provides complete protection to reinforcements tested for long durations under wet/dry treatments with mortars in saline water and laboratory atmospheres. Electrochemical impedance spectroscopy, direct current cyclic polarization, polarization resistance, and visual observations are used for quantitative and qualitative evaluations of the protective effects of the tested additives. X-ray diffraction analysis, scanning electron microscopy, and energy-dispersive X-ray spectroscopy analysis of the corrosion products formed on the embedded steel surfaces help explain the possible mechanisms behind the considerable improvement in the inhibitive effects of a mixed composition of borate and silicate. This combination also improves the compressive strength and workability of the mixed concrete. The results reveal that the synergistic protection provided by a mixture of borate and silicate can be attributed to the co-deposition of an iron-boron + ferrosilicate + cortensitite (an iron-silicon phase) film on the rebar surface.

## Introduction

The chloride-induced uniform and pitting corrosion of steel reinforcement bars embedded in concrete poses severe concerns in terms of the lifespan and safety of structures. Various methods and materials have been proposed and used to control the distressing effects of the localized and uniform corrosion of embedded steel rebars on concrete. Among the different techniques used to mitigate corrosion, the blending of additives in ready-to-cast concrete structures is considered to be economical and user friendly. The majority of commercially available additives for concrete used to control chloride-induced corrosion are primarily nitrite- and/or amine-based formulations^1–3^. The findings of different researchers regarding the performance integrity of such additives are contradictory and cast doubt on the long-term performance of the additives, particularly under harsh exposure conditions^4–6^. To overcome the limitations of traditional additives, researchers are focusing their attention on developing blends for concrete with little effect on its basic structure to achieve the ultimate goal of controlling the chloride-induced uniform and localized corrosion of rebars and improving the mechanical properties of cast structures. Owing to the massive weight and volume of concrete used in the construction of various structures, economical, eco-friendly, sustainable, and lightweight materials need to be adopted to control the corrosion of embedded steel reinforcement bars. Because the setting and hardening of concrete involve complex chemical reactions, the presence of foreign materials in concrete may have adverse effects on the long-term strength of structures. Therefore, a protective device that is compatible with the composition of concrete and mortars should be designed. The use of additives in concrete to formulate protective devices is a natural choice that can mitigate concerns regarding undesirable effects on structures long after their construction. Silica in different forms and alkaline ingredients are two components that comprise a major fraction of different types of cast concrete and mortar. Alkalinity plays a decisive role in the nucleation and growth of passive films on the surface of steel rebar. A higher total alkalinity results in a stable and protective passive film on the surface of steel rebar in contact with concrete. Silica reacts with the calcium hydroxide in Portland cement to form a calcium silicate hydrate gel that effectively reduces the porosity of hardened concrete and imparts strength. The direct inhibitory effect of silicate ions on rebar corrosion is limited based on the poor solubility of calcium silicate in the concrete components in pore solutions. Silicate ions added to concrete have been reported to indirectly affect the durability of steel reinforcement bars by modifying the structure of concrete and creating a tortuous path for the diffusion of chlorides, sulfates, and acidic gases. The presence of such ions in concrete has also been reported to act as an autonomic self-healing agent^7^, alkali activator in alkali-activated cements^8^, setting accelerator and waterproofing agent in paints and coatings^9,10^, microstructure and strength enhancer for water-rich materials^11^, and strength booster for cured concrete^12–14^. Information regarding the direct role of silicate ions in metal corrosion is also available as many researchers have reported the inhibitory effects of silicate ions on steel corrosion in alkaline environments^15–24^. Synergistic combinations of two or more chemicals are developed for concrete to get improved inhibition against corrosion and pitting of embedded rebars, enhanced mechanical properties of cast concrete, improved workability and reduce the cost.^25–30^. Silicate ions in combination with rare earth metal ions have been reported to receive a synergistic boost in terms of inhibiting the corrosion of steel exposed to an aerated NaCl solution^31^. In combination with polyamidoamine dendrimers, such ions are also known to have a synergistic protective effect against steel corrosion in soft water^31–33^. In addition to iron-based alloys, silicates have been reported to impart corrosion inhibition to other metals such as aluminum, zinc, and lead. Properties such as low weight, innocuous environmental effects, low cost, and good compatibility with concrete components have motivated many researchers to evaluate the effect of silicates on the corrosion inhibition of steel rebar in chloride-contaminated simulated concrete pore solutions^34,35^.

Boron-containing compounds such as borates and boric acid control the corrosion of steel embedded in concrete^36,37^. However, many researchers have reported either negligible or, in many cases, deteriorating effects of boron-based compounds added to concrete on the properties of concrete and reinforcement bars^38–41^. The inhibitory effects of boron-based compounds on iron and steel can be attributed to their pH-buffering action^42–46^. A review of the literature reveals that many researchers reported no formation of silicate or borate phases on the surfaces of rebar exposed to concrete containing silicate and borate compounds, respectively.

Surface analyses of steel rebar extracted from normal concrete generally do not reveal the formation of any compounds containing silica and iron, indicating that the silica present in cement or gravel is insensitive to interactions with the iron on the surface of rebar. The protective properties of the passive films formed on steel rebar surfaces in different types of concrete remain largely consistent. While studying the role of the extraneous addition of inorganic ions to concrete on their protective effects against the chloride-induced corrosion and pitting of steel rebar, we noted that a combination of sodium silicate and sodium borate dramatically improved protective properties. This motivated us to perform a detailed study to optimize the composition of a synergistic mixture of these two salts, analyze their mechanisms of action, and understand their role in the compressive strength of cast cubes. In this communication, we report the effects of simple silicate compounds, namely sodium silicate and sodium tetraborate, on the chloride-induced corrosion of steel reinforcement bars embedded in mortar. We found that a small concentration of these additives improves the compressive strength of cured mortars, increases the workability of wet concrete mixtures, and has a pronounced effect in terms of controlling the uniform and pitting corrosion of reinforcement bars caused by chloride diffusion in concrete. This communication presents the results of our study with an elaborate discussion on the mechanisms of the synergistic protection imparted by the combination of borate and silicate against the chloride-induced corrosion of steel reinforcement bars.

## Experimental details

### Testing materials

The thermomechanically treated steel rebar with a diameter of 16 mm used in this study had the following chemical composition:

$$ {\text{C}} = 0.{31};{\text{ Si}} = 0.{22};{\text{ Mn}} = 0.{86};{\text{ S}} = 0.0{1};{\text{ Cr}} = 0.0{1};{\text{ P}} = 0.0{3};{\text{ Ni}} = 0.0{3};{\text{ Cu}} = 0.0{4}. $$

All elemental concentrations are given in terms of weight percentages.

The bar had an approximately 2-mm-thick tempered martensite rim around its outer diameter, whereas the core was pearlite ferrite. To remove mill scale and rust from the rebar surface, the rebar was abraded on a motorized wheel fitted with 200-grit emery paper. Prior to placing the rebar in the corrosion cells and mortar, its surface was swabbed with moist ethanol tissue paper to ensure the removal of any oil or dust.

### Design of corrosion cells to evaluate rebar in a simulated concrete pore solution saturated with lime (SPSL)

**Figure 1:**
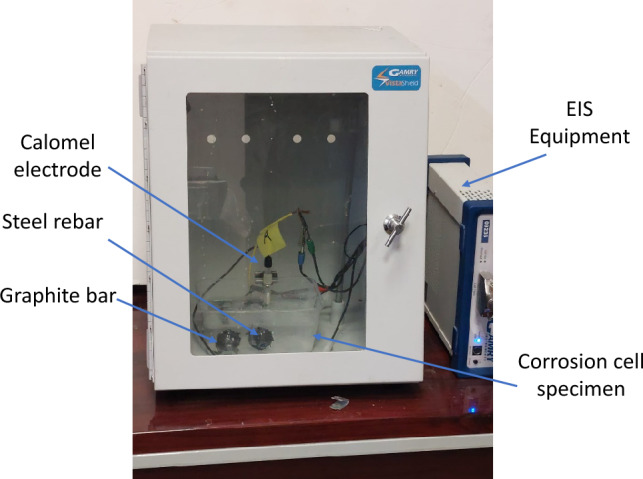
Electrochemical cell used to conduct experiments in SPSL.

To conduct electrochemical impedance spectroscopy (EIS) and DC polarization experiments, descaled and abraded rebar with a length of 150 mm was fitted into the electrochemical cell presented in Fig. 1. Two graphite rods with diameters of 10 mm and the same length as the bar were also fitted horizontally in the corrosion cell. These rods were short-circuited by a copper wire and used as auxiliary electrodes for electrochemical experiments. The test rebar was placed 20 mm away from the rods in the cell, as shown in Fig. 1.

The purpose of placing the two graphite rods in the corrosion cell was to ensure adequate surface area for the auxiliary electrode. The ends of the graphite rods and rebar emerging from the test cell were coated with epoxy resin to prevent the cell from leaking. The simulated concrete pore solution, which is described in “Composition of the simulated pore solution”, was poured into the electrochemical test cell for our experiments. The appropriate leads of the potentiostat cable were connected to the graphite rod and rebar ends outside the cell. A saturated calomel electrode was used as a reference electrode.

### Design and composition of mortar embedded with rebar

**Figure 2:**
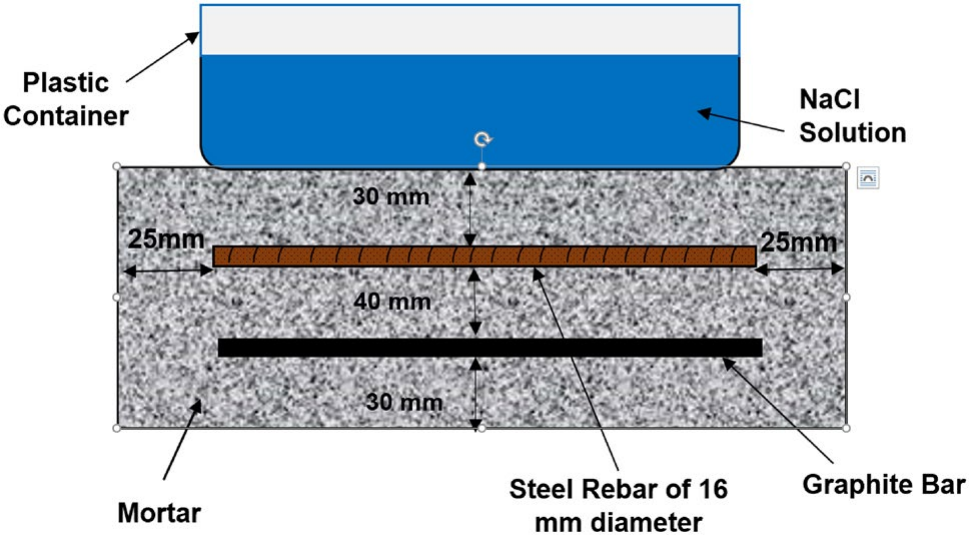
Schematic of the mortar used in this study.

To evaluate the effects of the additives on rebars embedded in mortars, abraded and de-oiled rebars were placed into mortars schematically shown in Fig. 2.

To prevent the counter electrode geometry from affecting the polarization data, graphite rods with the same diameter as the rebar (16 mm) were cast in the mortar (Fig. 2) and placed parallel to the steel rebar. The graphite rods were used as a counter electrode during our electrochemical studies. To avoid crevice corrosion at the two rebar ends, 15 mm sections at both ends were coated with epoxy and Teflon tape. The insulted copper wire was tightly wrapped around the surface of the rebar and graphite rods prior to applying the tape and coating. The wires protruding from the mortar acted as electrical leads and were connected to the potentiostat. Of the 150 mm length of the rebar samples, only 120 mm was exposed to the mortar, so a 25 mm cover thickness was available for the steel bars from all casting sides. Four sets of mortars with and without additives were mixed as follows.

- Control mortars: Control
- Mortars blended with sodium tetra borate: Borate
- Mortars blended with sodium silicate: Silicate
- Mortars blended with sodium tetra borate and sodium silicate: Borate + Silicate

The mortars were demolded 24 h after casting and cured in a humidity chamber maintained at 95% RH and at 25 °C until the age of 28 days. Ordinary Portland cement conforming to the specifications of ASTM 150C Type 1, water, and sand (mixed in proportions of 1:0.5:2) were used to cast the mortars. The sand particle size was within the range of 0.5–1.00 mm. Casting and curing of the specimens were performed according to the ASTM-C192 standard.

### Composition of the simulated pore solution

The protective properties of borate, silicate, and their combinations were initially studied by adding them to a simulated pore solution and performing electrochemical experiments on rebar after 15 d of exposure to the pore solution. This period of exposure was selected to ensure the formation of a passive film and minimize changes in open-circuit potential during the experiments. The pore solution composition was as follows^47,48^.

The following salts were added to doubly distilled water (mol/L): NaOH = 0.10, KOH = 0.30, Ca(OH)_2_ = 0.03, gypsum = 0.002, NaCl = 1.0. After vigorous mixing, the solution was stored in an airtight plastic container to prevent carbonation. After pouring the solution into the test cell, the cell mouth was closed with a lid. The pH of the prepared solution measured at 27 °C was 13.6 ± 0.05.

Based on the results of rapid tests in the chloride-containing simulated pore solution, the optimized composition of additives was determined with respect to the weight of the cement used to cast the mortars. This composition provided solid protection for the rebar surfaces embedded in the mortar.

### Composition of tested additives

The most synergistic composition of additives was determined by performing EIS tests on rebar exposed to chloride-containing simulated pore solutions blended with different concentrations of silicate and borate ions as their sodium salts. The optimized concentration of the additives was blended into the aforementioned solution and different electrochemical tests were conducted on the exposed rebar.

### Testing procedure

#### Wet/dry treatment of mortars

Wet/dry treatment of the mortars accelerated the corrosion rate of the embedded rebar. The test procedures used to assess the performance of rebar embedded in mortar have been described in previous papers^49,50^. The rebar-embedded mortars were subjected to wet/dry treatments (10 days wet in 5% sodium chloride solution and 20 days dry in a laboratory environment). The wet/dry treatments of the rebar embedded in the mortar continued for up to 43 cycles. After 43 treatment cycles, bleeding spots were observed on the surfaces of the control mortars. EIS tests were conducted at this stage on all mortars. The samples were then broken to observe the embedded rebar surfaces and digital images were recorded. Approximately 0.5 g of the corrosion products formed on the surface of the rusted rebar was scraped off and stored in airtight plastic pouches for further investigation. Because no rust spots were observed on the surface of the rebar embedded in borate + silicate mortars, small pieces were cut from this rebar and stored in airtight plastic pouches for further study.

#### Electrochemical experiments

EIS and polarization tests were conducted on the rebar exposed to pore solutions and embedded in mortars, as described in our previously published papers^49,50^. EIS studies were performed by applying a sinusoidal voltage of 10 mV at the open-circuit potential of the working electrode while changing the frequency from 100 kHz to 0.01 Hz. The obtained EIS data were analyzed using the CMS-300 software (M/S Gamry Instruments). DC polarization experiments (polarization resistance, anodic polarization and cyclic polarization) were conducted according to the procedures described in ASTM standards^51^ and detailed in previous publication^52^. The scan rate of potential for all DC polarization experiments was kept at 0.166 mV/second. The reference and auxiliary electrodes were saturated calomel electrode and graphite rods. All the electrochemical tests were performed at room temperature (25 ± 2 °C).

#### Compressive strength, workability, and setting time

The compressive strengths of the mortar specimens were determined according to the ASTM C109 standard. Mortar cubes were cast by adding the optimized concentration of additives, as described in “Composition of tested additives”, based on the dry weight of the cement. The cubes were demolded after 24 h and maintained at 95% RH until the date of testing. Six mortar cubes were tested after 28 and 90 days. The average results for each additive are presented and discussed in the results section. The workability and setting times of the mortars were evaluated according to the ASTM C1437 standard.

### Characterizations of corrosion products

- X-ray Diffraction (XRD)
  XRD studies were performed using a Siemens D-500 XRD system with a Cu-Kα (λ = 1.54 Å) radiation source. Scans were performed from 10° to 90° with a scan rate of 3°/min.
- Scanning electron microscopy (SEM) and energy-dispersive X-ray spectroscopy (EDX)
  SEM and EDX analyses of the corrosion products formed on the surfaces of the rebar embedded in the mortars were conducted using a Nova NanoSEM-450 device.

## Experimental results

### Electrochemical corrosion studies on rebar exposed to the chloride added simulated pore solution

Use of simulated pore solutions provide quick and reproducible results on the corrosion resistance of corroding interfaces. The evaluated electrochemical parameters for rebars exposed in such solutions although vastly differ from those derived for mortars embedded rebars, the trend of change in parameters by and large remain same under both the test conditions^52^. In view of this the optimization of the concentrations of two components of the tested inhibitors (borate and silicate) were carried out in the above-mentioned simulated pore solution (as stated above at “Composition of the simulated pore solution”). One mole of NaCl which is equivalent to 0.6 M Cl^-^ was added in pore solution before pouring it in the corrosion cells fitted with rebars. Thus, no time was allowed for pre-passivation of the exposed rebars. This ensured the most possible aggressive test environment for the optimization of the tested inhibitors. The inhibitor system optimized under such a harsh condition is expected to protect rebars under actual field conditions polluted with different concentrations of chloride.

#### Optimization of additive concentrations based on EIS tests

**Figure 3:**
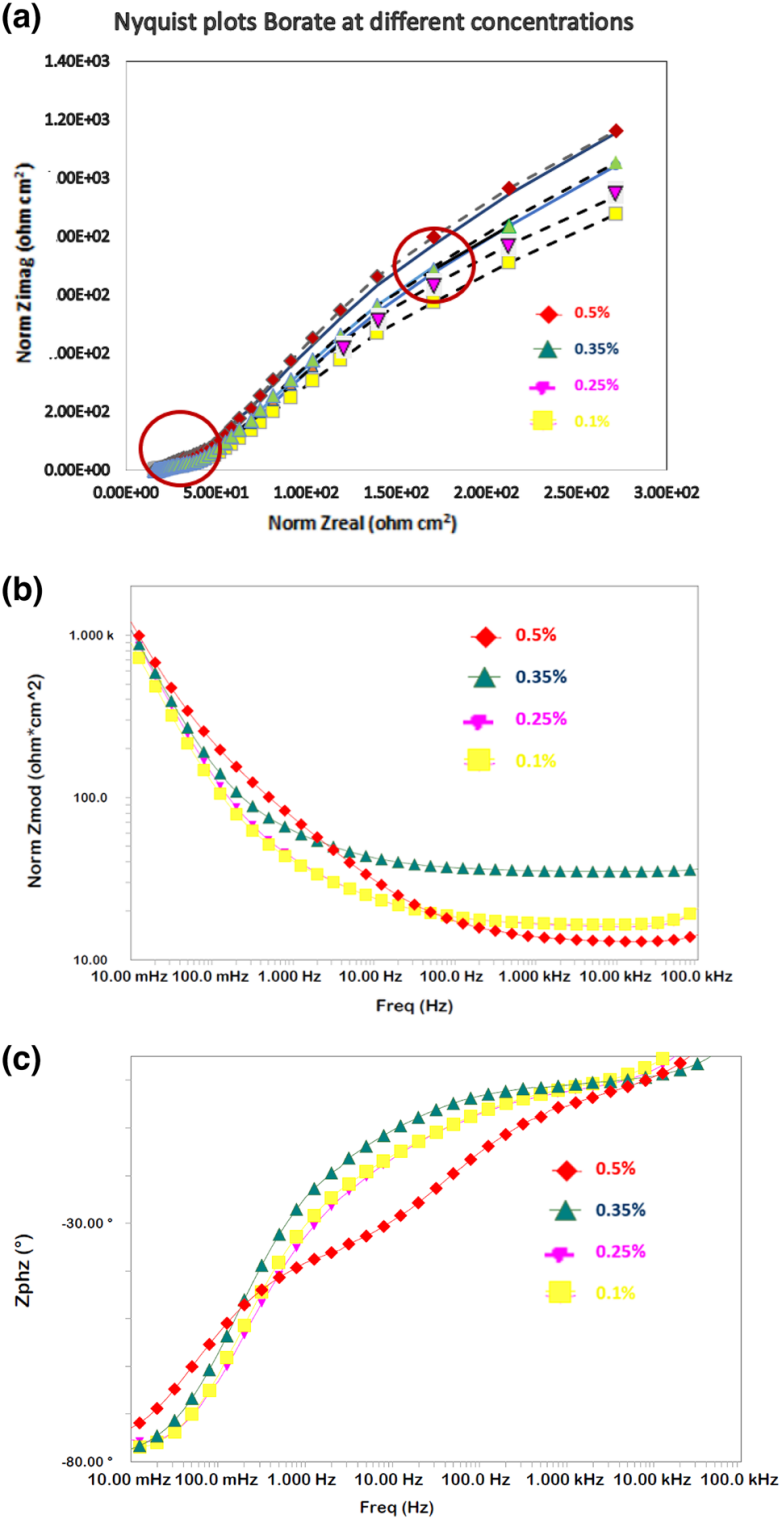
(**a**): EIS Nyquist plots for rebar exposed to the pore solution with different concentrations of added borate tested after 15 days. (**b**): EIS Bode log frequency versus log-normalized impedance plots for rebar exposed to the pore solution with different concentrations of added borate after 15 days. (**c**): EIS Bode log frequency versus phase shift plots for rebar exposed to the pore solution with different concentrations of added borate tested after 15 days.

**Figure 4:**
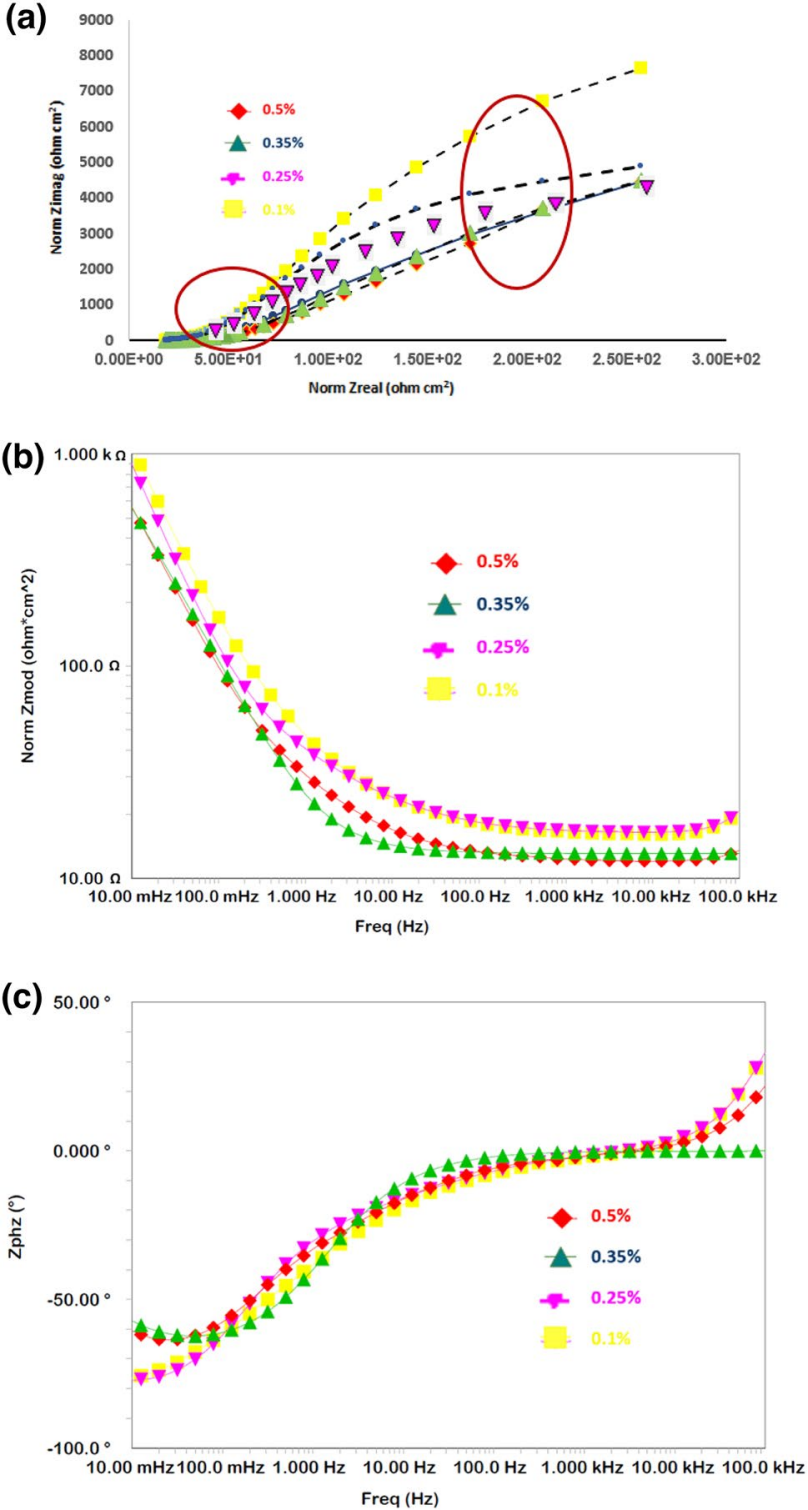
(**a**): EIS Nyquist plots for rebar exposed to the pore solution with different concentrations of added silicate after 15 days. (**b**): EIS Bode log frequency versus normalized impedance plots for rebar exposed to the pore solution with different concentrations of added silicate after 15 days. (**c**): EIS Bode log frequency versus phase shift plots for rebar exposed to the pore solution with different concentrations of added silicate after 15 days.

Our initial studies indicated that a combination of borate and silicate provided synergistic protection against the uniform and pitting corrosion of rebar in chloride-containing pore solutions. To obtain the optimal concentrations of these two additives, a screening test was conducted by performing EIS experiments with different concentrations of borate and silicate separately, as well as their combination, added to chloride-blended SPSL. Impedance behaviors in the form of Nyquist and Bode plots are presented in Figs. 3 and 4 for different concentrations of borate and silicate. The plots for only four concentrations (0.10%, 0.25%, 0.35%, and 0.50%) among the studied compounds are presented in these figures as Nyquist (Fig. 3a) and Bode plots (Fig. 3b,c). The Nyquist plots indicate the presence of two-time constants at all the four studied concentrations of the borate additive. The first one is at the higher frequencies (at about 10^5^ Hz) and the other at intermediate frequency range of 10–50 Hz, indicated by circles in the plots. Due to severe distortions, the Nyquist plots did not appear in the form of perfect semi circles. The features noted in Nyquist plots at the highest frequency is always part of semi-circle representing the charge—transfer resistance in parallel with the double layer capacitance, apart from the solution resistance^53^.

The nature of the plots at different concentrations of borate is generally consistent, except for variations in impedance at the lowest studied frequency as seen in Bode plot (Fig. 3b). This impedance at the lowest studied frequency, which is also known as Z_max_, is related to the corrosion resistance of the corroding interface. In this figure, one can see that the Z_max_ for the borate compound increases with its concentration and is maximized at a concentration of 0.5%. Above this concentration, its addition did not improve Z_max_ (plots not shown). The log-frequency-phase shift plots in Fig. 3c also exhibit similar characteristics for all four studied concentrations of this compound.

In contrast to borate, the silicates in the pore solution exhibited different behaviors at different concentrations (Fig. 4a–c).

In this case also the Nyquist plots exhibit two maxima as encircled in the plots and the compressed arc for 0.1% silicate is bigger than that noted at other concentrations of this compound. In Bode plots (Fig. 4b the maximum value of Z_max_ can be observed at a concentration of 0.1% for this compound. At higher concentrations, Z_max_ is noted to decrease. The curves for frequency-phase shift plots present identical nature at all the four concentrations of silicate.

**Figure 5:**
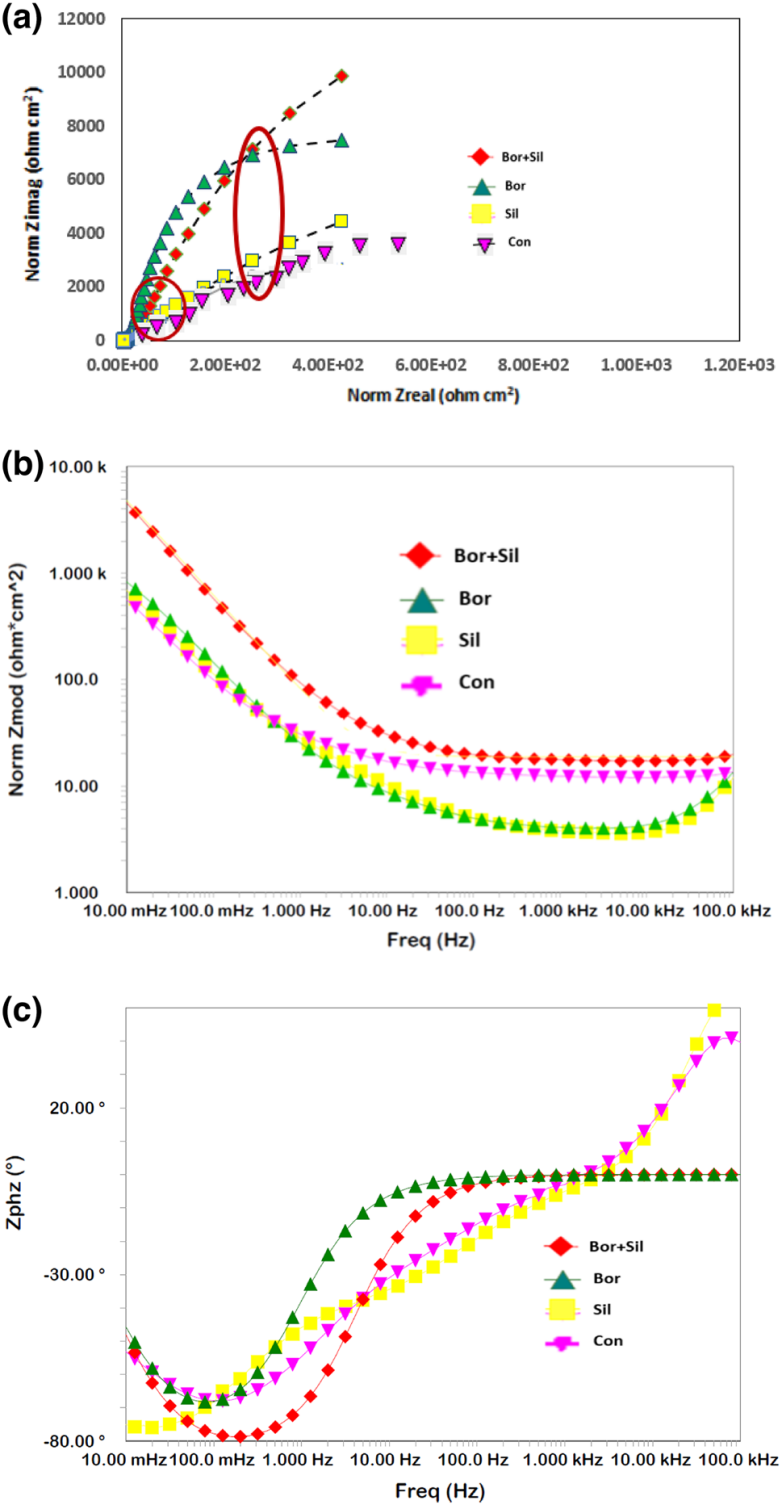
(**a**): EIS Nyquist plots for rebar exposed to the control and blended additive pore solutions after 15 days. (**b**): EIS Bode log frequency versus log normalized impedance plots for rebar exposed to the control and blended additive pore solutions after 15 days. (**c**): EIS Bode log frequency versus phase shift plots for rebar exposed to the control and blended additive pore solutions after 15 days.

To test the synergistic protective effect of a mixture of borate and silicate, the optimal concentrations (borate: 0.50%, silicate: 0.10%) were added to the chloride-containing pore solution and EIS tests were conducted after 15 d of rebar exposure (Fig. 5a–c). The Nyquist plots shown in Fig. 5a also exhibits two time constants around the same frequencies as observed for the Figs. 3a and 4a (indicated by circles). The arc for optimized mixture of borate + silicate is bigger than the control and individual components i.e. silicate and borate. This indicates that the combined mixture provides synergistic protection for the corroding interface. The value of Z_max_ for the combination of borate and silicate is considerably greater than the individual components and that for the control (Fig. 5b).

**Figure 6:**
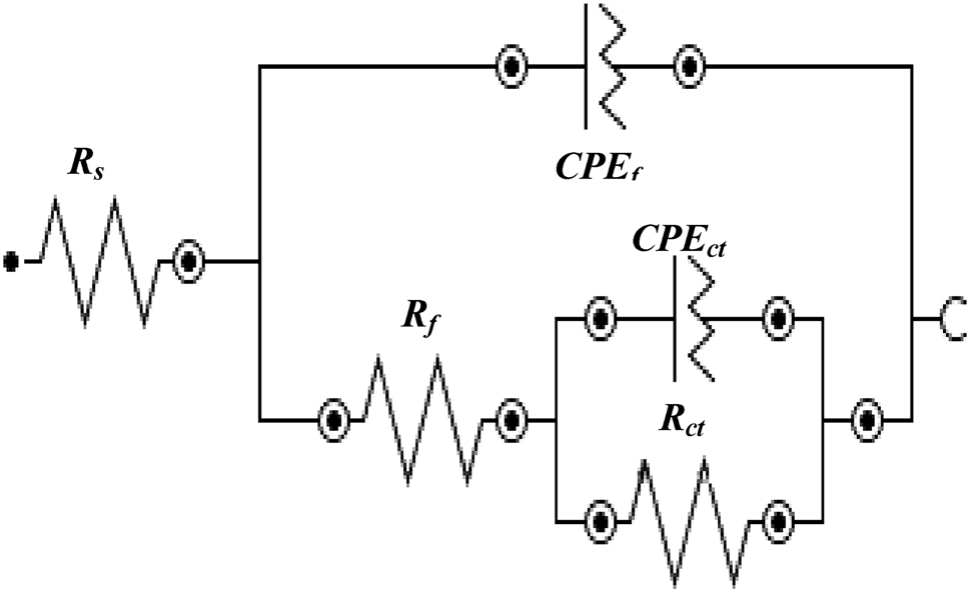
Schematic presentation of the electrical components associated with reactions occurring on the rebar surface.

To obtain quantitative information regarding the protective effect of the additives, results were extracted by fitting the experimental data in an appropriate simulated equivalent electrical circuit. Several permutations and combinations of components for the electrical elements revealed that a circuit consisting of series resistance (R_s_) related to the solution ionic resistance, two parallel RC circuits represented by two constant-phase elements (CPE), and charge-transfer resistance (R_ct_) (see Fig. 6) provided the best fitting results with the smallest error. Such circuits are used for fitting of the EIS data where meal surface is covered with oxide film^54–56^. The CPE indicates surface inhomogeneity^57^, which was expected in these tests based on the formation of corrosion products on the steel surface. This element is an empirically derived mathematical description of experimental impedance data and is defined as^58^

1 $$ Z = {1}/{\text{Y}}_{{\text{o}}} {\text{x}}\left( {{\text{j}}\omega } \right)^{ - \alpha } . $$

here, Y_o_ contains capacitance information and α is an empirical constant related to the characteristics of the CPE. The value of α may vary between zero and one depending upon the nature of the corroding interface. If the interface behaves as a pure resistor, then the value of α is zero. If it behaves as a pure capacitor, then α is equal to one^59,60^. In the above circuit, one CPE at higher frequencies represents the surface oxide film-electrolyte interface and the other at lower frequencies corresponds to the charge transfer resistance taking place at the metal-electrolyte interface. The fitted curves for borate and silicate are presented respectively in Figs. 3a–c, 4a–c and 5a–c. In these plots the marker points are experimental data and solid/dashed lines are for the fitted ones. The quantitative data extracted from the impedance plots presented in Figs. 3 and 4 are recorded in Table 1.

**Table 1:** Electrochemical parameters extracted from the impedance plots of Figs. 3 and 4 using simulated electrical circuit shown in Fig. 6, for different concentrations of borate and silicate.

% additives	Borate						Silicate					
	*R*_*film*_ (kΩ.cm^2^)	*Y*_*film*_ (S.s^a^/cm^2^) (1 × 10^−6^)	*α*_*film*_	*R*_*ct*_ (kΩ.cm^2^)	*Y*_*ct*_ (S.s^a^/cm^2^) (1 × 10^−6^)	*α*_*ct*_	*R*_*film*_ (kΩ.cm^2^)	*Y*_*film*_ (S.s^a^/cm^2^) (1 × 10^−6^)	*α*_*film*_	*R*_*ct*_ (kΩ.cm^2^)	*Y*_*ct*_ (S.s^a^/cm^2^) (1 × 10^−6^)	*α*_*ct*_
0.10	1.32	1109	0.87	6.65	544	0.81	1.60	1490	0.87	3.32	911	0.76
0.25	1.90	987	0.88	7.00	508	0.86	1.56	1504	0.88	3.04	987	0.75
0.35	4.65	432	0.91	12.89	32.06	0.89	1.45	1607	0.89	3.12	956	0.76
0.50	4.67	407	0.84	12.08	30.87	0.88	1.54	1509	0.93	3.13	989	0.74

The R_film_ values for the film formed on the surface of rebars inhibited with borate is noted to increase with its concentration, with the highest value at the concentration of 0.5%. The Y_film_, on the other hand is observed to decrease with concentration. The R_ct_ and Y_ct_ also follow the same trend except that the values for these parameters respectively increase and decrease to a greater extent. For silicate, the R_film_ is greater at the concentration of 0.1% than that noted at the higher concentrations. No considerable change is noted for the value of Y_film_ of this additive. A similar trend is also recorded for the parameters R_ct_ and Y_ct_. These results again suggest that the optimum concentration for borate and silicate are respectively 0.5% and 0.1%.

A negligible effect of increasing the concentration of silicate in improving the corrosion resistance of the interface is attributed to its effect in impeding the oxidation of Fe^2+^ to Fe^3+^^61^. It is known that the formation of maghemite (λ-Fe_2_O_3_) on the surface of steel rebars exposed in simulated pore solution results in the development of stable passive film^48^. The presence of silicate in the pore solution hinders the oxidation of initially ionized Fe^2+^ in to maghemite (λ-Fe_2_O_3_) and hence impedes the formation of stable passive film on the surface of rebars.

The quantitative data extracted from the impedance plots in Fig. 5 using the simulated circuit presented in Fig. 6 are listed in Table 2. A distinct difference can be observed in the protection imparted by the synergistic mixture of the studied compounds compared to the control condition. The charge transfer resistance for the corrosion of rebar exposed to the chloride-containing pore solution blended with the synergistic mixture is approximately eight times greater (26.22 KOhm·cm^2^) than that of the control sample (3.22 KOhm·cm^2^) tested under identical conditions. The parameter Y_ct_ which is admittance and indicates the ease of electrodic reactions at the corroding interface is significantly low for mixture of the components (bor + sil) than the control sample. These results further confirm that the two studied additives when tested under mixed conditions effectively increase the protective action.

**Table 2:** Electrochemical impedance parameters extracted from the impedance plots in Fig. 5 using the simulated electrical circuit presented in Fig. 6.

Impedance parameters											
Control						Borate + silicate					
*R*_*film*_ (kΩ.cm^2^)	*Y*_*film*_ (S.s^a^/cm^2^) (1 × 10^−6^)	*α*_*film*_	*R*_*ct*_ (kΩ cm^2^)	*Y*_*ct*_ (S.s^a^/cm^2^) (× 10^−6^)	*α*_*ct*_	*R*_*film*_ (kΩ cm^2^)	*Y*_*film*_ (S.s^a^/cm^2^) (× 10^−6^)	*α*_*film*_	*R*_*ct*_ (kΩ cm^2^)	*Y*_*ct*_ (S.s^a^/cm^2^) (× 10^−6^)	*α*_*ct*_
1.11	230	0.88	3.22	135	0.87	4.11	15.22	0.89	26.22	9.21	0.86

#### Effect of additives on anodic polarization

**Figure 7:**
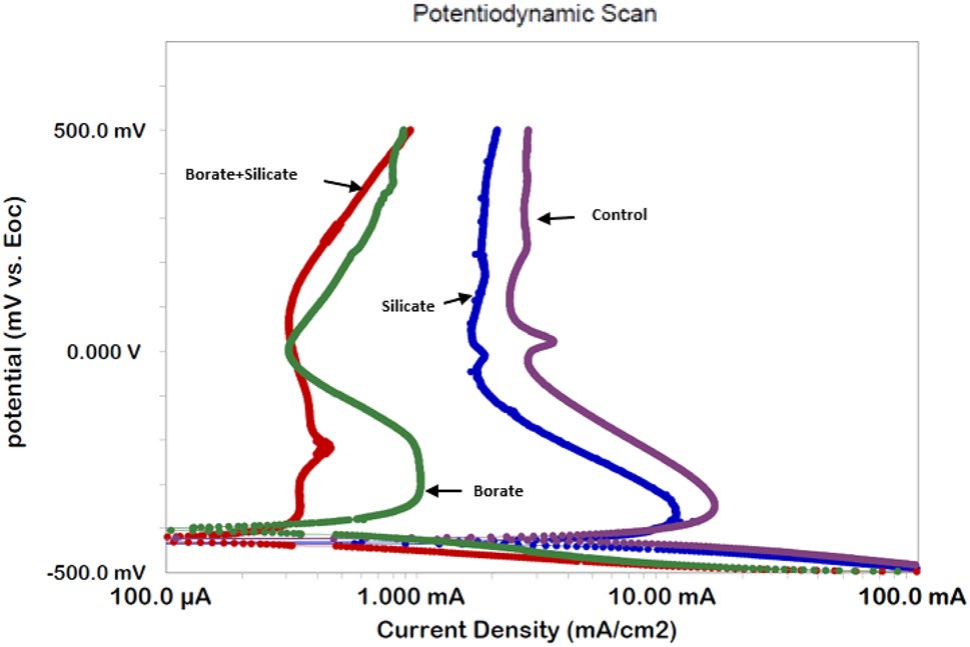
Potentiodynamic polarization of rebar exposed to the control and blended additive pore solutions after 15 days.

Polarization studies provide crucial information regarding the corrosion behavior of a corroding interface. Our experiments were conducted by exposing the rebar to chloride-containing pore solutions blended with different additives and the results are presented in Fig. 7. Some common and distinct features can be observed in these plots.

Active–passive behavior above the open-circuit potentials can be observed for both the control and blended additive solutions. The plot for the synergistic mixture exhibits very stable passivity and the curve is significantly shifted toward the low-current–density region compared to the control condition and individual additives. These results corroborate our EIS findings, where the synergistic mixture of borate and silicate imparted higher protection than the control condition and individual compounds.

#### Control of pitting tendency

**Figure 8:**
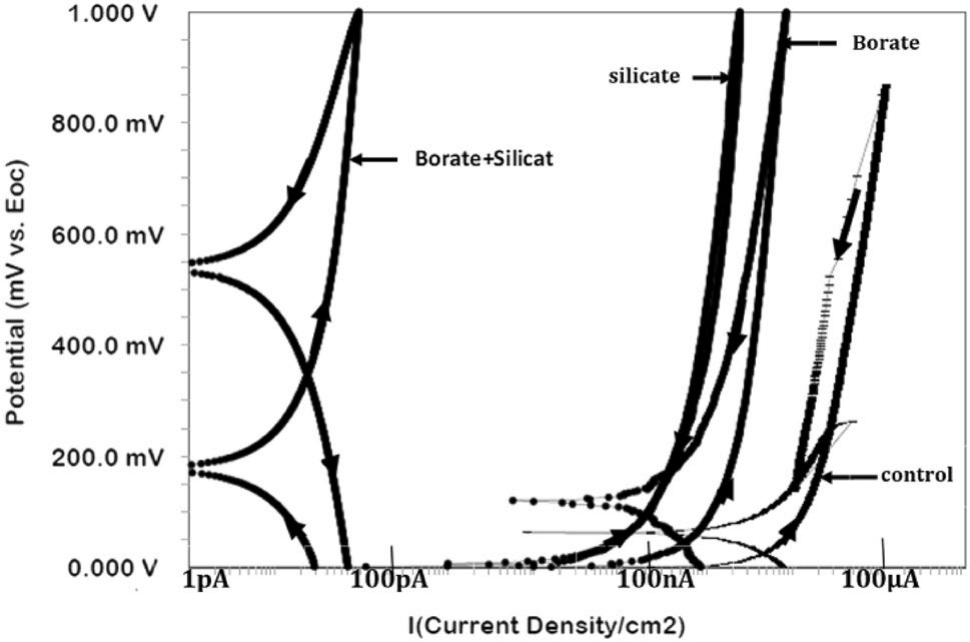
Cyclic polarization of rebar exposed to the control and blended additive pore solutions after 15 days.

Cyclic polarization experiments were conducted to assess the pitting tendency at the corrosion interface. Cyclic polarization plots for the control condition and additives are presented in Fig. 8. Forward and backward scans are indicated in each plot. The pitting tendency of a corroding interface is typically assessed based on the positive loops of such plots and their breakdown potentials. In Fig. 8, one can see that the breakdown of the passive film did not occur in any of the tested cases, indicating that the combined effect of the added chloride and imposed potential was not sufficient to break the passive film formed at the corroding interface. However, these conditions undoubtedly affected the stability of the films to varying degrees, as indicated by the current loops formed in the plots associated with different additives. The control sample exhibits the largest loop, followed by silicate and borate, and the smallest loop can be observed for the synergistic mixture of borate and silicate. These findings suggest that the optimized synergistic mixture is effective at strengthening the passive film, even under the influence of chloride contamination and anodic polarization.

### Electrochemical results for rebar embedded in mortars

**Figure 9:**
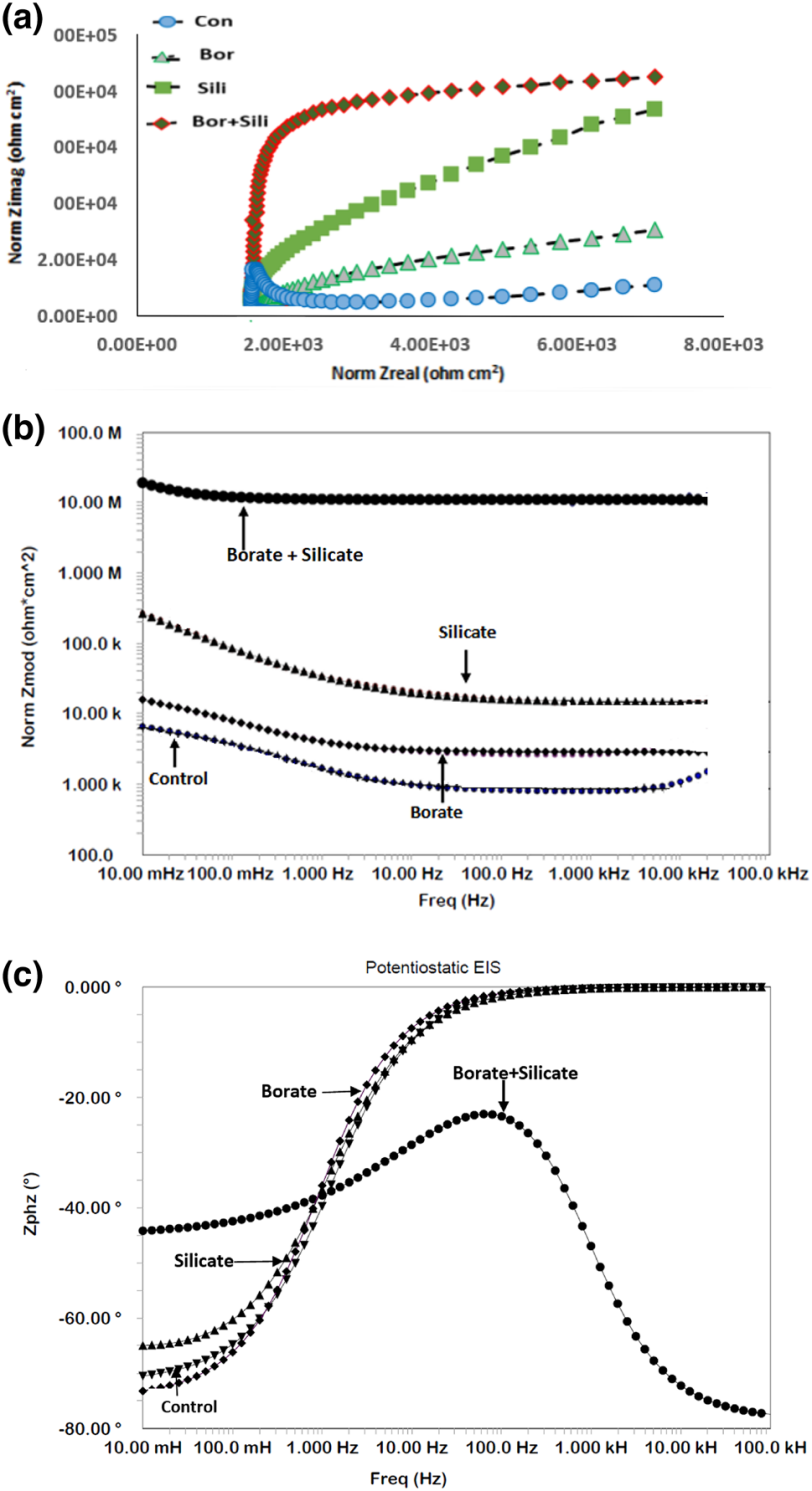
(**a**): Nyquist impedance plots for the control and blended additive mortars over 43 cycles of wet/dry treatments. (**b**): Electrochemical frequency-impedance plots for the control and blended additive mortars over 43 cycles of wet/dry treatments. (**c**): Electrochemical frequency-phase shift plots for the control and blended additive mortars after 43 cycles of wet/dry treatments.

**Figure 10:**
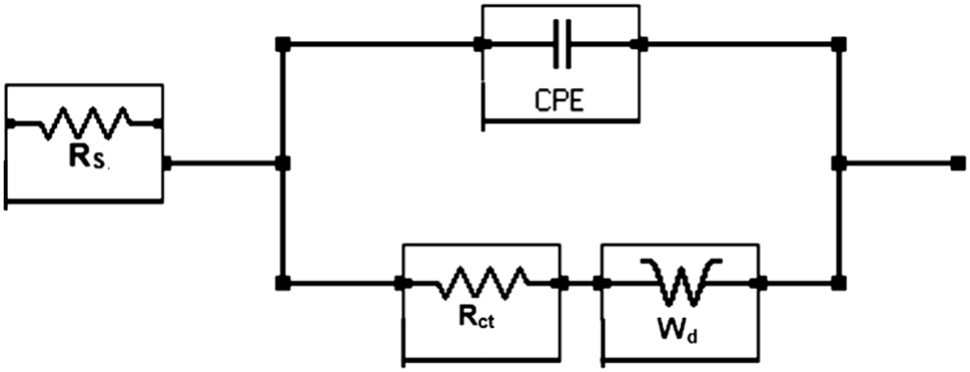
Equivalent electrical circuit used to extract the impedance parameters by best fitting method.

Electrochemical studies of rebar exposed to mortar are closer to the conditions of real applications than submersion in synthetic pore solutions. To obtain an accurate representation of the role of each additive, the mortar casts detailed in the experimental section were subjected to wet/dry treatments and tested at different intervals. Figure 9a–c present the impedance plots for a control sample and mortars with additives incorporated at the concentrations optimized during our study of rebar exposed to the chloride-containing simulated pore solution, after 43 cycles of wet/dry treatments. The nature of these plots differs significantly from that of the plots recorded for exposure to the simulated pore solution. The Nyquist plots (Fig. 9a) at low frequency significantly deviate from -1 slope normally expected for a system showing semi-infinite Warburg impedance. The curve for borate + silicate at low frequencies is parallel to real impedance axis. Such plots are attributed to the presence of interfaces with semi-infinite and finite space Warburg impedance^62–64^. The above figures reveal that a very stable passive film was formed on the surface of the rebar embedded in the mortar blended with optimized borate and silicate content. The best fit with the smallest error with chi square values of the order of × 10^−4^ was observed when the Warburg diffusion element was added to the constant-phase element circuit, shown in Fig. 10. This indicates that some tortuous paths developed on the surface of the corroding rebar embedded in the mortar. This is unsurprising because the rebar exposed to the mortars experienced longer durations of wet/dry treatments (43 cycles); thus, sufficient time was available for the nucleation and growth of the passive film. The extracted data with least error using the equivalent electrical circuit is presented in Table 3.

**Table 3:** Electrochemical parameters of corroded specimens embedded in mortars with different additives after 43 cycles of wet/dry treatments as determined using EIS.

Sample ID	R_s_ (KOhm cm^2^)	R_ct_ (KOhm·cm^2^)	Y_0_ (S·s^α^/cm^2^) × 10^−6^	α	W_d_ (S × s^1/2^/cm^2^ × 10^−6^)	% IE	χ^2^ × 10^−4^
Control	1.20	5.33	287	0.64	2.6	–	2.1
Borate	1.45	12.50	174	0.51	2.9	57.36	4.2
Silicate	14.23	90.70	151	0.56	2.7	94.12	9.6
Borate + Silicate	18.43	1120.00	4.0	0.53	6.8	99.52	1.9

From the above plots we can see that the passive film on the surface of the rebar achieved significant stability, as indicated by the Z_max_ values and computed data recorded in Table 3. Another interesting feature in the impedance plots presented in Fig. 9b is the superior performance of silicate compared to borate, which contradicts our observations for the simulated pore solution.

The data recorded in Table 3 reveal that the charge transfer resistance of the synergistic mixture (Borate + Silicate) is much greater than those of the control condition and individual compounds. The admittance values (Y_0_), which are also related to the nature of the corrosion interface, are considerably lower for the mixed additives compared to the control condition and constituent compounds. These results suggest that the addition of borate and silicate to mortars imparts a very high degree of protection to rebar subjected to chloride-induced corrosion. The Warburg component (W_d_) for inhibited mortars is higher than the control mortar. This element relates to diffusivity of oxidant and reductant as well as oxygen and chloride ions across the corroding interface^51,65^.

These findings suggest that the mixture of the optimized concentrations of the inhibitor forms very stable surface film on the surface of mortars embedded rebars and effectively protect them from the corrosive effect of chloride, moisture and oxygen. To confirm the superior performance of the synergistic mixture of borate and silicate ions over the control condition and individual compounds, the mortars were subjected to polarization resistance tests after 43 cycles of wet/dry treatments using the linear polarization technique. The resulting data are listed in Table 4. We can see that the combination of borate and silicate provides almost complete protection (inhibition efficiency of 99.99%) against the chloride-induced corrosion of rebars.

**Table 4:** Electrochemical parameters of corroded specimens after 43 cycles of wet/dry treatments as determined by the linear polarization resistance method.

Sample ID	R_p_ (KOhm·cm^2^)	Corrosion rate (µm/year)	% Inhibition efficiency
Control	0.178	25.25	–
Borate	0.777	5.8	77.00
Silicate	8.671	0.52	97.00
Borate + Silicate	6.46 × 10^3^	0.0007	99.99

**Figure 11:**
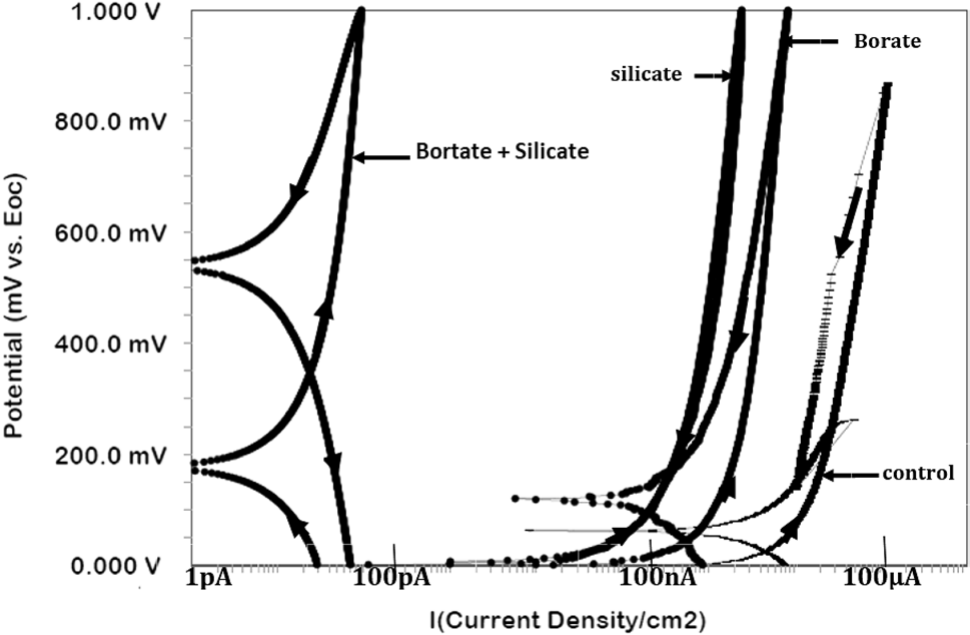
Cyclic polarization curves for rebar embedded in mortars blended with different additives after 43 cycles of wet/dry treatments.

The rebars embedded in mortars were also subjected to cyclic polarization. The polarization plots are presented in Fig. 11. The forward and backward potential scans are indicated by arrows on the curves. The generated current for the rebar protected by the combined borate and silicate is in the picoampere range, indicating a very high degree of protection against corrosion.

### Compressive strength, setting time, and flowability

The flowability, early and delayed setting strengths (28 and 90 days, respectively), and initial and final setting times were evaluated for the control condition, individual compounds (at different concentrations), and optimized mixture. The results are recorded in Table 5. The data in this table reveal that after 90 days of curing, the addition of borate to the mortars at all concentrations significantly improved the compressive strength compared to the control mortar. The flowability and setting time also increased significantly. These results corroborate the findings of the other authors who reported increased setting time without any adverse effect on compressive strength of sodium silicate—alkali activated slag cement concrete mixed with sodium tetraborate^66–69^ . In contrast, silicate ions have a deteriorating effect on strength, except at a concentration of 0.1%. The optimized composition (0.5% borate, 0.1% silicate) beneficially affects the compressive strength (after 90 days of curing), improves the fluidity of the mixture, and increases the setting time. All of these modifications are beneficial for field operations.

**Table 5:** Compressive strength after 28 and 90 days of aging, flowability, and initial and final setting times. The control sample had a cement–water-sand ratio of 1:0.50:2. The additives were added to the control composition of the mortar as weight percentages of the amount of cement.

ID	Composition	Flowability (%)	Comp. strength (MPa)		Setting time (min)	
			28 days	90 days	Initial	Final
1	Control	101	55	59	155	270
2	0.1% borate	102	50	72	140	300
3	0.25% borate	113	48	53	165	340
4	0.5% borate	113	48	64	285	360
5	0.75% borate	86	43	62	260	360
6	1.0% borate	117	49	66	35	360
7	0.1% Silicate	101	51	72	190	260
8	0.25% Silicate	109	53	61	160	255
9	0.5% Silicate	111	51	56	160	245
10	0.75% Silicate	118	50	55	160	255
11	1% Silicate	123	46	57	160	250
12	0.1% borate + 0.1% Silicate	91	54	71	140	280
13	0.25% borate + 0.1% Silicate	116	60	67	210	315
14	0.5% borate + 0.1% Silicate	122	54	64	310	360
15	0.75% borate + 0.1% Silicate	120	49	56	360	360
16	1% borate + 0. 1% Silicate	120	42	47	100	360

### Morphology of corrosion products

**Figure 12:**
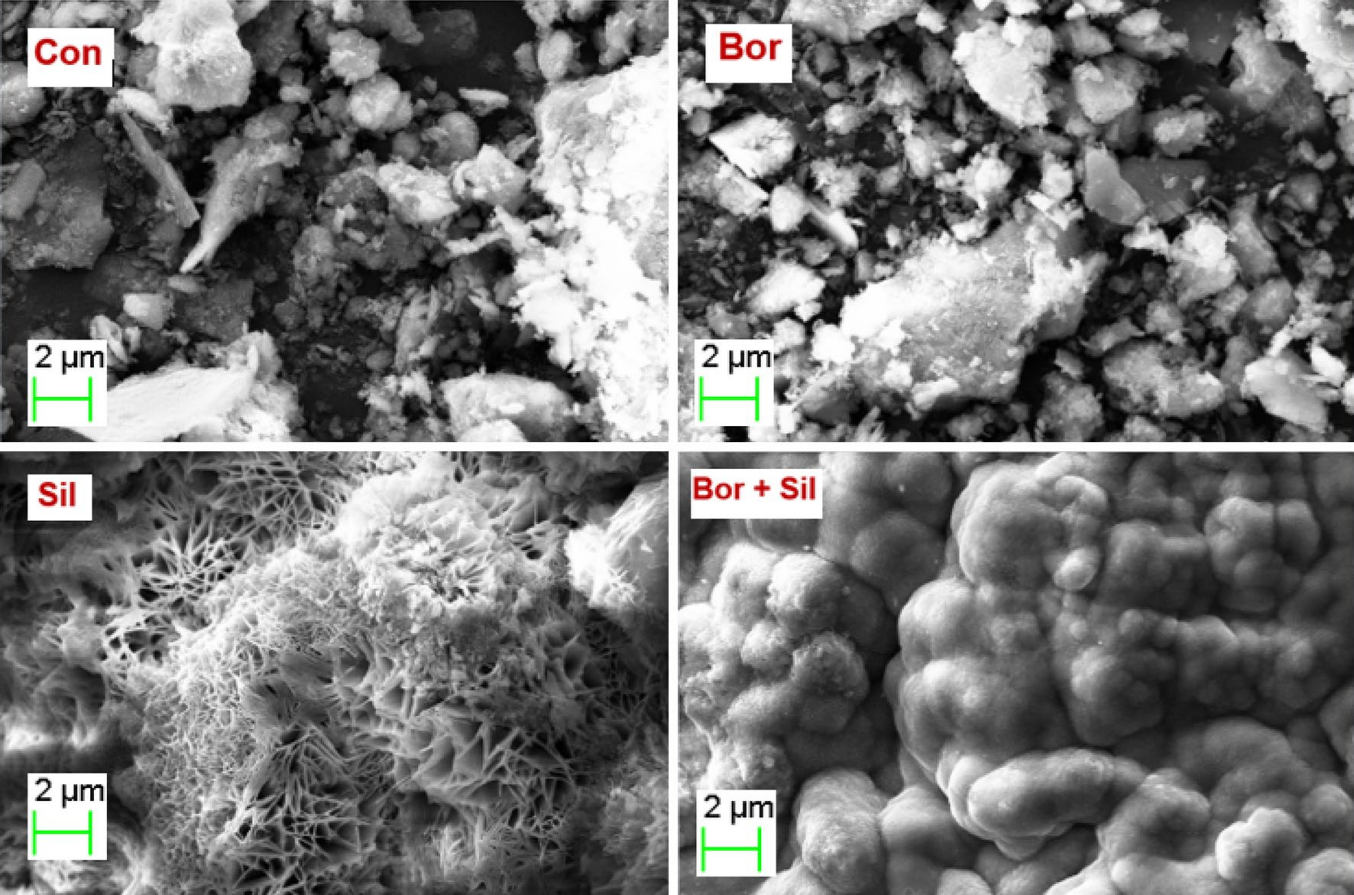
Scanning electron microphotograph of the film formed on the surface of rebar embedded in the control (Con), Borate (Bor), Silicate (Sil) and Borate + Silicate (Bor + Sil) mortars.

Scanning electron microphotographs of the corrosion products formed on the surfaces of the rebar in the control mortar and mortars with additives retrieved after 43 cycles of wet/dry treatments are presented in Fig. 12. The morphology of the corrosion products formed on the surface of the control rebar is characterized by a haphazard deposition of cylindrical, rod-shaped, and tubular-shaped particles across the surface (Fig. 12). This type of morphology is attributed to the presence of the akageneite (β-FeOOH) phase of rust^70^. The morphologies of the corrosion products formed on the rebar surfaces embedded in the borate-containing mortar are characterized by the dense deposition of particles. The rust morphology on the surface of the rebar embedded in the silicate-containing mortar exhibits bird nest shapes. In the case of the synergistic composition, the film on the rebar surface exhibits a very tightly bonded globular deposition.

### XRD analysis of corrosion products

**Figure 13:**
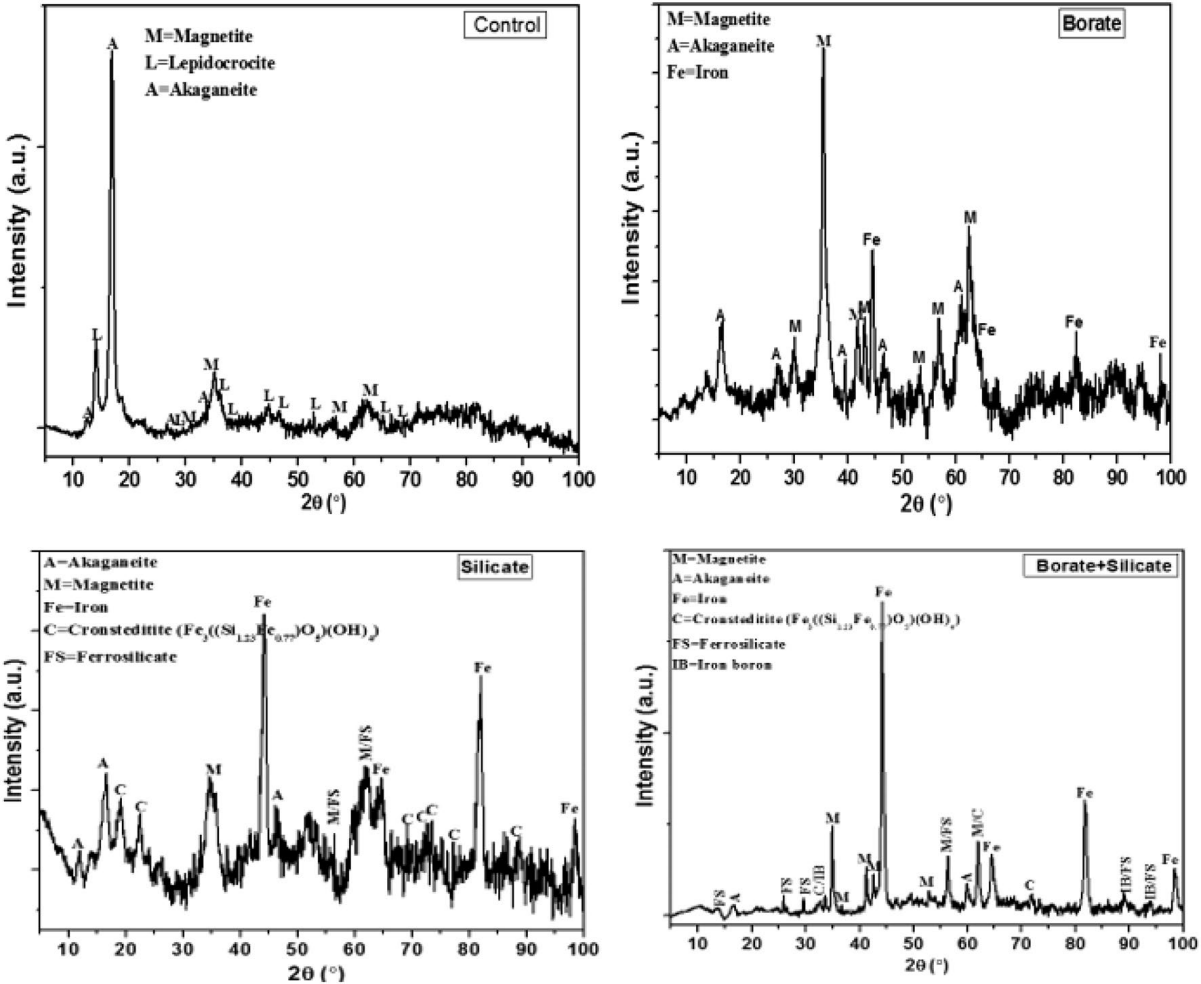
XRD spectrum of the film formed on the surface of rebar embedded in mortars.

The XRD patterns of the corrosion products formed on the rebar surfaces embedded in the control and additive-blended mortars are presented in Fig. 13. A very strong akageneite peak with smaller magnetite and lepidocrocite peaks can be observed for the corrosion products of the control (con) rebar (Fig. 13). The presence of stronger akageneite peaks in the corrosion products of iron and steel indicates that chloride ions migrated and accumulated to a significant level at the corroding interface.

This conclusion is supported by the EDX analysis results in Table 6. The chloride content of the rust in the control rebar was 4.52%. In the akageneite phase of rust, the chloride level is as high as 6%^71^. Such a high level of chloride and the presence of a very strong XRD peak for akageneite indicates that the alkalinity of the pore solution at the corrosion interface of the control solution was reduced by the wet/dry treatment cycles. Although an akageneite phase was present in the rust on the rebar embedded in the mortars with additives, in comparison to the control rebar, the corresponding peaks are significantly weaker (Fig. 13). In the borate-inhibited rebar rust, akageneite, magnetite, and iron phases were detected. In the silicate and silicate + borate synergistic mixtures, multiple phases of rust, including akageneite and magnetite, were observed. Two new phases, namely consteditite (Fe_3_((Si_1.23_Fe_0.77_)O_5_)(OH)_4_) and ferrosilicate, appeared in the silicate rust, and the borate + silicate synergistic mixture significantly inhibited the formation of rust. Peaks of iron and boron also appear in the results for the mixed composition of borate + silicate.

**Table 6:** EDX results for the films formed on the surfaces of rebar embedded in the control, borate, silicate and borate + silicate mortars.

Mortar mixtures	Element, (weight %)				
	O	Cl	Ca	Si	Fe
Control	32.38	4.52	1.49	0.64	60.97
Borate	35.66	1.66	1.37	0.65	60.66
Silicate	37.39	0.36	1.37	0.95	59.93
Borate + Silicate	28.32	0.11	1.12	1.15	69.30

### Digital images of rebar removed from mortars after 43 cycles of wet/dry treatments

**Figure 14:**
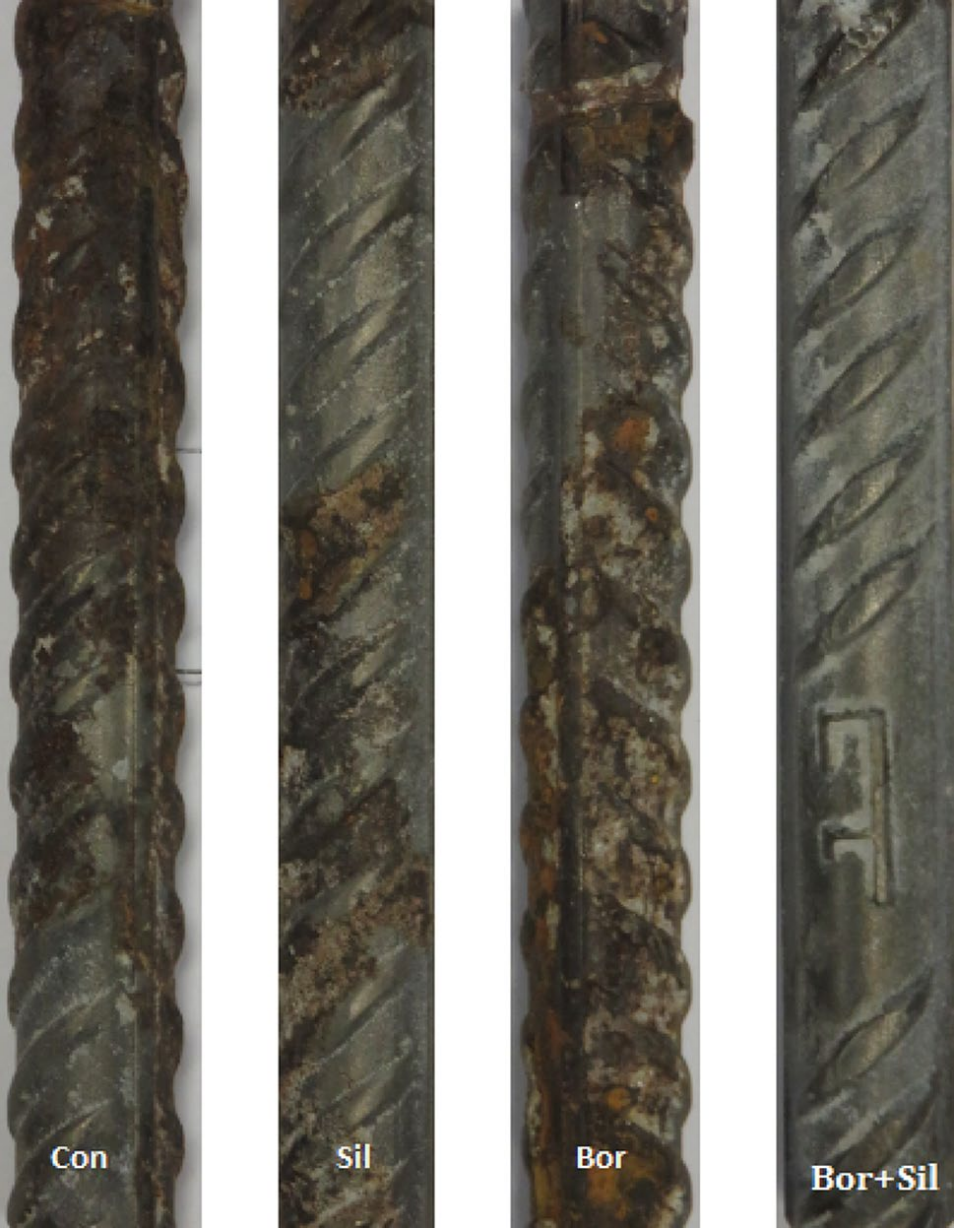
Images of rebar samples embedded in different mortars (Con = control; Sil = silicate; Bor = borate; Bor + Sil = borate + silicate) and removed after 43 cycles of wet/dry treatments.

Digital photographs of rebars retrieved from the mortars after 43 wet/dry treatment cycles are presented in Fig. 14. The control mortar rebar is severely rusted and pitted. The silicate- and borate-inhibited rebar also appears to have deteriorated during the wet/dry treatments. Pitting damage can also be observed in some locations. In contrast to the individual compounds, the synergistic mixture resulted in rebar that appears to be clean, free of rust, and unaffected by chloride. These observations corroborate the findings and inferences drawn from the other techniques described in the previous sections.

## Discussion

The results described in the previous sections indicate that silicate added individually to concrete imparts reasonably good corrosion protection. However, in combination with borate, it provides nearly complete protection against the chloride-induced corrosion of rebar. Electrochemical studies indicated that silicate addition can provide an inhibitive protection efficiency of at least 97% against the chloride-induced corrosion of rebar embedded in mortars after 43 cycles of wet/dry treatments, whereas under identical test conditions, borate alone yielded an efficiency of only 77% (Table 4). When combined at optimal concentrations, borate and silicate provide an inhibitive protection efficiency on the order of 99.99% (Table 4). It is worth to discuss the significant improvement in protection for silicate addition in mortars whereas very little benefit was noted when added in pore solution (Table 1). As stated in preceding paragraphs, a negligible effect of increasing the concentration of silicate is attributed to its effect in impeding the oxidation of Fe^2+^ to Fe^3+^^72^. This resulted in inhibition of formation of maghemite (λ-Fe_2_O_3_) on the surface of steel rebars exposed in simulated pore solution which is responsible for stable protective passive film^48^. The presence of silicate in the pore solution hinders the oxidation of initially ionized Fe^2+^ in to maghemite (λ-Fe_2_O_3_) and hence impedes the formation of stable passive film on the surface of rebars. It appears that its enhanced protection in mortars is related to a different mechanism than that described above for the synthetic pour solution. In mortars the silicate had greater role in densifying the pour structures of the cast mortars. This effect is evident from the data for parameter Rs recorded in Table 3. The value of R_s_ for silicate added mortar is about 10 times higher than the borate added mortar. Apart from the pour solution resistance, R_s_ also provides information on compactness of pores of mortars. The XRD results presented in Fig. 13 provide further clues on beneficial role of silicate in combination of borate in boosting the inhibition effects. The XRD spectra show that two common phases related to silicate, namely ferrosilicate and cronsteditite (Fe_3_SiO_5_(OH)_4_), were formed on the surface of the rebar inhibited by silicate, as well as by the synergistic mixture (Fig. 13). These results suggest that silicate plays a decisive role in controlling the chloride-induced corrosion of rebar. Based on these observations, it is important to discuss the role of silicate in boosting the inhibitory effects of borate. Among silica-based additives in concrete, silica fume is the most common. In particular, silica fume is a popular material for achieving pozzolanic effects in concrete. It is commonly understood that when this pozzolanic material is added to concrete, it improves compactness and compressive strength, and reduces porosity, chloride content, moisture content, and oxygen diffusivity. In addition to these improvements, some researchers have indicated that adding silica fume to concrete has a strengthening effect on the passive films formed on embedded rebar. It has been suggested that this effect can be attributed to the partial solubility of silica in the pore solution, which forms orthosilicate anions^73^. Orthosilicate anions are large multivalent ions that have a strong tendency to adsorb preferentially onto steel and steel oxidation products in place of hydroxide and chloride ions^74,75^. It has been suggested that the adsorption of silicates onto iron and iron oxides results in the formation of ferrosilicate^76^, which is insoluble in acidic and alkaline solutions. The co-deposition of ferrosilicate and iron oxides onto steel surfaces has been reported to protect against corrosion more effectively than iron silicate alone^75^. XRD analysis of the corrosion products deposited on silicate-inhibited rebar revealed the presence of ferrosilicate, cronsteditite, and iron oxide phases, namely magnetite and akageneite (Fig. 13), which corroborates the theory described above. These two iron oxide phases were also present on the rebar surface and were inhibited by the synergistic mixture. We observed a stable phase of iron-silica minerals with a large negative value of the standard Gibbs free energy of formation (Δ_f_G^0^ =  − 2613 kJ/mol)^76^. Among the other two phases (ferrosilicate and cronsteditite), cronsteditite has not been previously reported to form on steel surfaces exposed to neutral or acidic aqueous solutions. However, in a strongly alkaline solution (10 M KOH) blended with sodium silicate, Cekerevac et al. reported the formation of this iron-silicon complex phase on a steel surface^77^. This finding corroborates our findings, suggesting that environmental alkalinity plays a decisive role in the formation of cronsteditite. Cronsteditite forms as a result of the aqueous dissolution of silicates, generating aqueous SiO_2_ in solution^78,79^. The overall reaction for the formation of cronsteditite can be written as

2 $$4Fe+SiO2\left(aq\right)+7H2O \to \left({\text{Fe}}3{\text{SiO}}5\left({\text{OH}}\right)4\right)+10 H2.$$

This equation suggests that the aqueous silica content in the solution is the main constituent controlling the formation of cronsteditite. Temperature, pH, and ionic strength significantly affect the formation of aqueous SiO_2_^80^. The addition of borate to an aqueous solution containing silicate promotes the solubility of silica in proportion to its concentration in the solution^81^. When added to concrete, this compound plays a dual role. First, it increases the alkalinity and solubility of silica^82^. Both of these factors favor reaction (2) for the formation of cronsteditite. The enhanced corrosion protection provided by the synergistic mixture of borate and silicate is likely a result of the boosting effect of borate on the formation of cronsteditite on the steel surface. Additionally, borate itself is an anodic inhibitor of steel in alkaline solutions that forms a thin protective layer of iron-boron phases on steel substrates. The detection of an iron-boron phase in the XRD peaks of the borate + silicate-inhibited rebar (Fig. 13) indicates that the combined effect of the cronsteditite + iron boron phase boosted the inhibition of the chloride-induced corrosion of the rebar. Interestingly, no iron-boron phase was detected on the surface of the borate-inhibited rebar. This indicates that the addition of silicate to concrete facilitates the formation of a protective film of iron-boron.

## Conclusion

A mixture of optimized concentrations of borate and silicate materials imparted a very high degree of protection against the chloride-induced corrosion of steel rebars embedded in concrete construction buildings. Electrochemical studies revealed that this combination of additives significantly increased the charge transfer resistance of rebars, particularly after longer durations of exposure. XRD and SEM results revealed that protective films of iron silicate and cronsteditite, which are very stable phases of iron-silicon compounds, uniformly precipitated on the steel rebar surface and isolated the metal from the aggressive electrolyte. Mixing borate with silicate yielded a passive layer on the surface of rebars composed of iron-boron + cronsteditite + iron silicate. This optimized composition also improved the compressive strength of cast concrete after long curing durations (90 days) and the workability of wet concrete mixtures.

## Data Availability

All the datasets generated in the study are available on reasonable request from the corresponding author.
